# Supplementary material for: Morphometric Relationships, Growth and Condition Factors of Critically Endangered Chinese Pangolin (Manis pentadactyla)
Source: Animals (Basel). 2022 Apr 2;12(7):910. doi: 10.3390/ani12070910 (PMC8996938; doi:10.3390/ani12070910)
Supplement: Supplementary file 1 [file animals-12-00910-s001.zip › animals-1652932-supplementary.pdf]

Table S1. Descriptive statistics of total length (TL) and body weight (BW), LWRs and condition factors of Chinese pangolins by ages and sexes.

**Parameters**	**6 months old**		**1 years old**		**2 years old**		**3 years old and above**		**ANOVA(F-value)**	**Sig.**
	**Male**	**Female**	**Male**	**Female**	**Male**	**Female**	**Male**	**Female**		
**N**	44	38	76	47	22	20	25	10		
**TL (cm)**										
Min-Max	39-83.2	41.0 - 66.5	46-88	46-80	75.2-94	66-114.9	79.0-103.0	78.0-113.5	192.33	0.000
Mean+ SD	56.6 ± 8.4	55.9 ± 5.8	67.1 ± 8.5	65.3 ± 6.7	83.5 ± 4.1	80.1 ± 10.1	88.3 ± 6.0	87.0 ± 10.3		
**BW (kg)**										
Min-Max	1.0 - 1.9	0.9 - 1.9	2.0-3.9	2.0-2.9	4.0 - 4.9	3.0 - 3.9	5.0 - 7.6	4.0 - 5.8	556.46	0.000
Mean +SD	1.5 ± 0.3	1.6 ± 0.3	2.7 ± 0.6	2.4 ± 0.3	4.4 ± 0.3	3.5 ± 0.3	5.7 ± 0.7	4.6 ± 0.6		
**LWRs and Growth**										
a	0.13	0.1	0.02	0.44	3.21	2.29	0.91	2.43		
b	0.6	0.7	1.07	0.4	0.07	0.1	0.41	0.14		
Growth type	A-	A-	A-	A-	A-	A-	A-	A-		
r2	0.16	0.16	0.49	0.15	0.004	0.01	0.05	0.02		
Pearson Correlation	0.367*	0.372*	0.695**	0.405**	0.080	0.071	0.219	0.104		
**Condition Factors**										
**KF (g/cm3)**										
Min-Max	0.2-2.0	0.5 - 2.1	0.4-2.1	0.5-2.0	0.5 - 1.1	0.2-1.4	0.5 - 1.1	0.3-0.9	4.907	0.002
Mean+ SD	0.9 ± 0.4	1.0 ± 0.3	0.9 ± 0.3	0.9 ± 0.3	0.8 ± 0.1	0.7 ± 0.2	0.9 ± 0.2	0.7 ± 0.2		
**WC (kg)**										
Min-Max	1.3 - 2.2	1.3 - 1.9	1.7-4.1	1.5-2.1	3.7 - 6.7	1.9 - 3.6	5.7 - 7.8	3.4 - 4.0	190.418	0.000
Mean+ SD	1.7 ± 0.2	1.7 ± 0.1	3.0 ± 0.6	1.9 ± 0.1	5.8 ± 0.8	2.9 ± 0.7	6.5 ± 0.5	3.6 ± 0.2		

**KR**										
Min-Max	0.6-1.2	0.6-1.2	0.6-1.3	1.0 - 1.6	0.7 - 1.1	1.0 - 1.7	0.8 - 1.2	1.1 - 1.6	4.261	0.006
Mean +SD	0.9 ± 0.2	1.0 ± 0.2	0.9 ± 0.1	1.3 ± 0.1	0.8 ± 0.1	1.3 ± 0.2	0.9 ± 0.1	1.3 ± 0.1		
T-test (Sig.)	0.153		0.000		0.000		0.000			

N: Sample size, TL: Total length, BW; Body weight, SD: Standard deviation, a: intercept/co-efficient of LWRs, and b: slope of the equation, r2: regression co-efficient, -A: negative allometric growth, **: Significant(p<0.001), KF: Fulton condition factor, WC: Calculated weight, KR: Relative condition factor and Sig: p<0.005.

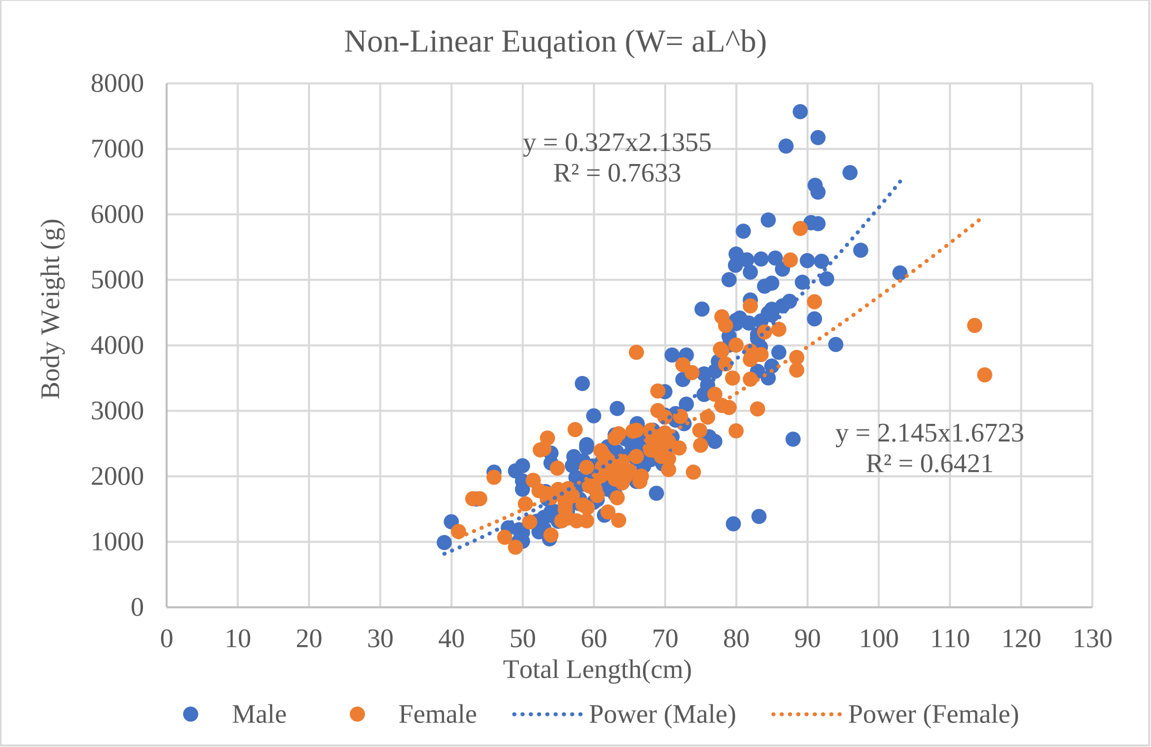

Figure S1. Length -weight relationship of Chinese pangolin by gender

Table S2. Length-weight relationships (LWRs) of Chinese pangolin in different months

Months	N	Mean ± SD		Intercept 'a'	Slope 'b'	Regression Co-efficient 'r'
		Total Length	Body Weight			
January	4	85.5 ± 4.2	5.7 ± 1.1	0.01	3.0	0.65
February	5	83.8 ± 10.1	4.6 ± 1.4	1.7	1.8	0.61
March	6		4.2 ± 0.8	0.02	2.7	0.79
April	6	87.0 ± 6.1	4.5 ± 1.2	0.03	2.6	0.44
May	9	81.2 ± 2.5	4.2 ± 0.9	0.09	0.9	0.0097
June	4	80.5 ± 3.7	4.4 ± 0.8	0.001	3.6	0.62
July	6	82.6 ± 4.9	4.8 ± 0.5	0.79	0.4	0.053
August	9	89.1 ± 13.0	4.5 ± 0.8	6.28	0.08	0.002
September	7	83.9 ± 16.3	4.4 ± 1.1	0.15	0.75	0.24
October	7	84.9 ± 3.5	5.0 ± 1.0	0.09	2.46	0.26
November	5	83.9 ± 6.1	4.9 ± 0.9	0.06	2.57	0.99
December	9	85.6 ± 6.6	4.9 ± 1.1	2.36	1.71	0.31

Table S3. Descriptive statistics of Fulton condition factor (K_F) calculated weight (W_C) and relative condition factor (K_R) in different seasons in adults.

Parameters	Seasons	N	Mean ± SD	SE	Cl 95%	t-test	Sig.
K_F	Dry	35	0.76 ± 0.14	0.03	-0.12 - 0.06	-0.697	0.488
	Wet	42	0.79 ± 0.22	0.03			

W_C	Dry	35	4.56 ± 0.67	0.11	-0.06-0.39	1.466	0.147
	Wet	42	4.40 ± 0.26	0.04			
K_R	Dry	35	1.03 ± 0.18	0.03	-0.09 - 0.08	-0.167	0.868
	Wet	42	1.04 ± 0.19	0.03			

Table S4. Linear and non-linear correlation between different parameters

**Correlations**	**Parameters**	**TL**	**BW**	**K_F**	**W_C**	**K_R**
Pearson's	TL	1	.837**	-.601**	.993**	0.026
	BW	.837**	1	-.162**	.835**	.519**
	K_F	-.601**	-.162**	1	-.562**	.729**
	W_C	.993**	.835**	-.562**	1	0.038
	K_R	0.026	.519**	.729**	0.038	1
Kendall's tau_b	TL	1	.695**	-.416**	1.000**	.102*
	BW	.695**	1	-.104**	.695**	.423**
	K_F	-.416**	-.104**	1	-.415**	.515**
	W_C	1.000**	.695**	-.415**	1	.103*
	K_R	.102*	.423**	.515**	.103*	1
Spearman's rho	TL	1	.866**	-.573**	1.000**	.125*
	BW	.866**	1	-.158**	.867**	.546**
	K_F	-.573**	-.158**	1	-.572**	.667**
	W_C	1.000**	.867**	-.572**	1	.125*
	K_R	.125*	.546**	.667**	.125*	1

TL: Total Length, BW: Body Weight, K_F: Fulton condition factor, W_C: Calculated weight, K_R: Relative condition factor, **. Correlation is significant at the 0.01 level and *. Correlation is significant at the 0.05 level.
